# Magnetic Fe_3_O_4_@Mg/Al-layered double hydroxide adsorbent for preconcentration of trace metals in water matrices

**DOI:** 10.1038/s41598-021-81839-8

**Published:** 2021-01-27

**Authors:** Luthando Nyaba, Tshimangadzo S. Munonde, Anele Mpupa, Philiswa Nosizo Nomngongo

**Affiliations:** 1 Department of Chemical Sciences, University of Johannesburg, Doornfontein Campus, P.O. Box 17011, Doornfontein, 2028 South Africa; 2 DSI/NRF SARChI: Nanotechnology for Water, University of Johannesburg, Doornfontein, 2028 South Africa; 3 DSI/Mintek Nanotechnology Innovation Centre, University of Johannesburg, Doornfontein, 2028 South Africa

**Keywords:** Analytical chemistry, Environmental chemistry, Inorganic chemistry, Materials chemistry, Surface chemistry, Chemical synthesis, Chemistry, Environmental sciences, Environmental chemistry

## Abstract

A magnetic Fe_3_O_4_@MgAl-layered double hydroxide (MLDH) nanocomposite was successfully synthesized and applied as an effective adsorbent for preconcentration of trace As(III), Cd(II), Cr(III), Co(II), Ni(II), and Pb(II) ions from complex matrices. The quantification of the analytes was achieved using the inductively coupled plasma optical emission spectrometry (ICP-OES) technique. The nanocomposite was then characterized using BET, FTIR, SEM, and EDS. Due to its high adsorption surface area, compared to traditional metal oxide-based adsorbents, MLDH nanocomposite exhibited high extraction efficiency. Several experimental parameters controlling the preconcentration of the trace metals were optimized using response surface methodology based on central composite design. Under optimum conditions, the linearity ranged from 0.1 to 500 µg L^−1^ and the correlation of coefficients (R^2^) were higher than 0.999. The limits of detection (LODs) and quantification (LOQs) were 0.11–0.22 µg L^−1^ and 0.35–0.73 µg L^−1^, respectively. The intra-day (n = 10) and inter-day precisions (n = 5 working days) expressed in the form of percent relative standard deviations (%RSDs) were below 5%. The proposed method was successfully applied for the analysis of the As(III), Cd(II), Cr(III), Co(II), Ni(II), and Pb(II) ions in different environmental water samples.

## Introduction

Determination and monitoring of toxic trace metals such as lead, cadmium, mercury, thallium, and arsenic, have received more attention in recent years^1–3^. This is because of their toxicity to humans and other living organisms as well as wide availability in water systems^1^. These metals come into contact with humans through food chain and uptake by plants^4^. They can accumulate in human organs such as lungs, liver, kidney, immune system, central nervous, and heart amongst others, thus leading to serious health effects^4,5^.

Numerous analytical methodologies have been used for the quantification of trace elements in water systems. These include flame atomic absorption spectroscopy (FAAS)^6^, atomic fluorescence spectrometry (AFS)^7^, inductively coupled plasma-mass spectrometry (ICP-MS)^8,9^ electrothermal atomic absorption spectroscopy (ETAAS)^10^, electrochemical methods, and ICP-OES. Among the aforementioned techniques, ICP-OES is one of the most widely used detection method^11^. This is because ICP-OES is robust and can be applied for the qualitative and qualitative analysis of multielement in complex matrices^12,13^. Therefore, owing to the low concentration of trace metals in various samples and the complexity of the sample matrices, there is a need for separation/preconcentration methods before ICP-OES determination. This is because the preconcentration step improves the LOD/LOQs, sensitivity, accuracy, and specificity of the analytical technique^14^.

Sample preparation methods such as dispersive liquid–liquid micro-extraction (DLLME)^15^, solid-phase extraction (SPE)^16,17^, dispersive solid-phase microextraction (DSPME), and dispersive magnetic solid-phase extraction (DMSPE)^18^, among others have been used for preconcentration of trace metals. Dispersive magnetic solid-phase extraction (DMSPE) is classified as a miniaturized SPE technique because smaller amounts of adsorbents are needed compared to the traditional SPE^19^. Other advantages of this technique include being easy to carry out, effective, and safe sample preparation method^12^. In this method, the adsorbent is added to the aqueous sample without conditioning step, instead, the sample with the sorbent is dispersed using external forces such as vortex, microwave, and ultrasonic bath, among others which improve extraction kinetics and adsorption process^20,21^. The adsorbent is then recovered by an external magnet and the analytes are desorbed using an acid^12^.

Design and choice of adsorbents in solid-phase based sample preparation techniques plays a most important factor to get a good recovery and high selectivity^13^. Among other adsorbents reported in the literature, nanomaterials have found great attention in recent years due to their high surface area and large adsorption area^22^. They are highly effective sorbents that can be applied for the pre-concentration of a wide range of analytes^23^. Nanomaterials such as layered double hydroxide (LDH) have received great attention in recent years due to their unique physical and chemical properties^24^. Layered double hydroxides are types of nanomaterials that are amongst a wide class of synthetic 2D anionic nano-clay materials^22^. The core aim of using LDH as sorbents over the other layered materials is because of its diversity and flexibility. As a result, LDH has been used as sorbents^25^, catalysts^26^, catalyst supports^27^, anion exchangers^25^, and molecular sieves^28^. Furthermore, LDH sorbents resemble mineral hydrotalcite (HT), a natural magnesium–aluminium hydroxyl carbonate (Mg_6_Al_2_(OH)_16_CO_3_·4H_2_O) because it consists of exchangeable adsorption sites. The attributes of LDH such as excellent anion exchange capacity, high surface area, good biocompatibility, specific structural characteristics, pH-dependent solubility, and high chemical stability are some of the few admirable properties which attracted several researchers’ interest^25,29^. While many magnetic LDHs have been synthesized, there are not many studies on their application as sorbents for pre-concentration of multi-element in complex matrices such as acid mine drainage and seawater.

The main purpose of this study was to develop an ultrasound-assisted-dispersive magnetic solid-phase extraction (UA-DMSPE) technique for preconcentration of trace quantities of As(III), Cd(II), Cr(III), Co(II), Ni(II) and Pb(II) ions from complex water matrices. Magnetic Fe_3_O_4_@Mg/Al-layered double hydroxide (MLDH) nanocomposite was used as an adsorbent for the extraction of selected trace metals. The response surface methodology (RSM) based on the central composite design (CCD) was employed to investigate the effects of important operating parameters such as sample pH, mass of adsorbent, eluent concentration, and extraction time on the preconcentration of trace metals.

## Experimental

### Reagents and solution

All reagents were of analytical grade unless specified and double-distilled deionized water was used during the experiments. Iron (III) trichloride hexahydrate (FeCl_3_·6H_2_O, molecular weight 270.30 g mol^−1^_,_ 99%), ammonium hydroxide solution (28%), iron(II) dichloride tetrahydrate (FeCl_2_·4H_2_O, molecular weight 198.81 g mol^−1^, 99%), magnesium nitrate hexahydrate (Mg(NO_3_)0.6H_2_O molecular mass 256.41 g mol^−1^, 99.999%), aluminium nitrate (Al(NO_3_)_3_ molecular weight 256.41 g mol^−1^, 99%), obtained from Sigma-Aldrich (St. Loius, MO, USA). A synthetic sample of multi-element at concentration of 50 μg L^−1^ was prepared from Spectrascan single element standards (1000 mg L^−1^) of As, Cd, Cr, Pb, Co, and Ni (Teknolab, Norway). A 100 mg L^−1^ Spectrascan multi-element standard solution (Teknolab, Norway,) for preparation working calibration standards. Certified reference material (ERM-CA713 trace metals in wastewater and standard reference materials (NIST SRM 1640a trace elements in natural water) were obtained from Sigma. NIST SRM 1643e was purchased from the National Institute of Standards and Technology (Gaithersburg, MD, USA).

### Instrumentation

Inductively coupled plasma–optical emission spectrometer (ICP-OES) (iCAP 6500 Duo, Thermo Scientific, UK) equipped with a charge injection device (CID) was employed for quantification of As(III), Cd(II), Cr(III), Co(II), Ni(II) and Pb(II) ions. The ultrasound-assisted preconcentration of trace metals was performed in a Scientech ultrasonic bath system with a frequency of 50 Hz and power of 150 W (Labotec, Midrand, South Africa). Scanning electron microscope (SEM) (JSM-6360LVSEM, JEOL Co., Japan) was used to examine the morphology Fe_3_O_4_@Mg/Al-LDH nanocomposite. The X-ray powder diffraction (XRD) patterns recorded using a Philips X-ray generator model PW 3710/31 a diffractometer with an automatic sample changer model PW 1775. The specific surface area, pore size, and pore volume values were measured using Surface Area and Porosity Analyzer (ASAP2020 V3. 00H, Micromeritics Instrument Corporation, Norcross, USA). Spectrum 100 FT-IR (PerkinElmer, USA) spectrometer equipped with Universal Attenuated Total Reflectance (ATR) was used to investigate the functional groups on the surface of the adsorbent, X-ray photon spectroscopy (XPS) on VG ESCALAB MARK II (VG, UK).

### Sample collection and preparation

The sampling of real water samples (tap and river water) was collected from south of Johannesburg, South Africa, and were kept in 1000 mL polypropylene bottles and stored in the fridge at 4 °C until analysis. Prior to analysis, the water samples were filtered using 0.25 µm acrodisc and syringe.

### Synthesis of Fe_3_O_4_ magnetic nano-particles

The synthesis of Fe_3_O_4_ was synthesized following the method reported previously^30^. Briefly, 10.1 g of FeCl_3_·6H_2_O and 5 g of FeCl_2_·4H_2_O salts were dissolved in 150 mL of deionized water. The reaction was carried out under vigorous stirring at 85 °C under a nitrogen atmosphere. Whilst continuing stirring, 25 mL aliquot of ammonium hydroxide solution was added dropwise into the solution to form a black precipitate. The mixture could stir at the same conditions for 15 min and then cooled at ambient temperature. The Fe_3_O_4_ was collected using an external magnet. The magnetic nanoparticles were rinsed with deionized water. The magnetitic nanoparticles were dried at 60 °C for 10 h in an over and then they were pulverized into fine particles using a pestle and mortar.

### Synthesis of Fe_3_O_4_@Mg/Al-LDH nanocomposite

The synthesis of Fe_3_O_4_@MgAl LDH nanocomposite was achieved following the procedure described elsewhere^7,31^. Briefly, 1.93 g of previously Fe_3_O_4_ nanoparticles were dispersed in 150 mL distilled water in a beaker for 15 min to obtain a uniform suspension. The precipitate formed was then stirred vigorously at 60 °C, whilst adding dropwise a mixture of 12.8 g Mg(NO_3_)0.6H_2_O and 9.38 g Al(NO_3_)0.9H_2_O solution. Then the pH was adjusted to 10 by adding a mixture of 6.75 g NaOH and 5.29 Na_2_CO_3_ solution. The precipitate was left to age for 8 h, then washed several times with distilled water and separated using a magnet. It was dried at 60 °C overnight.

### Preconcentration of trace metals using UA-DMSPE method

The ultrasonic-assisted dispersive solid-phase extraction procedure was carried according to our previous methods^12,32^. Briefly, 50–100 mg of the magnetic LDHs nanocomposite was weighed into 100 mL plastic bottles, then an aliquot of 20 mL synthetic sample solutions (pH 3–9) was added to the sample bottles. The extraction and preconcentration of the As(III), Cd(II), Cr(III), Co(II), Ni(II), and Pb(II) ions were carried out by ultrasonication for 5–30 min. After the pre-concentration process, the sample solution and nano adsorbent were separated using an external magnetic. The analytes desorbed by adding 2.5 mL of 0.1–1.0 HNO_3_ to the adsorbent and sonicated for 5 min. The eluent and the nanoadsorbent were separated by magnetic decantation. The aqueous phase containing the analytes was filtered into a pre-cleaned polypropylene centrifuge tube using the PVDF syringe membrane. The filtrate was then analysed by ICP-OES. The experimental parameters affecting the preconcentration method were optimized using RSM employing a CCD. These parameters include the mass of adsorbent (MA), extraction time (ET), sample pH; and eluent concentration (EC). Under optimal conditions, the above method was repeated for the validation and application of the proposed method.

### Method validation for the determination of trace metals

The performance and validation of the developed method were assessed using the linear range, precision (repeatability and reproducibility), preconcentration factor (PF), enhancement factor (EF), accuracy, the limit of detection (LOD), and limit of quantification (LOQ). The accuracy and precision (intraday and interday) were investigated using certified reference materials (ERM^®^-CA713) and standard reference materials (NIST SRM 1640a and 1643e). The accuracy was expressed as percentage recovery (%R) and relative error (%RE) and they were calculated according to Eqs. (1) and (2). The precision was expressed as relative standard deviation (%RSD) which was calculated using Eq. (3), where S_d_ is the standard deviation of five and ten replicates of the CRM analysis.

1 $$\% R = \frac{Obtained \,value}{Certified \,value} \times 100$$

2 $$\% RE=\frac{Obtained value-certified value}{Certified value}\times 100$$

3 $$\% RSD=\frac{{S}_{d}}{mean}\times 100$$

The LOD and LOQ were calculated as $\frac{3{S}_{d}}{m}$ and $\frac{10{S}_{d}}{m}$, where *S*_*d*_ is the Standard deviation of ten measurements of blank solution (analysed and *m* is the slope of the respective investigated trace metal ion calibration curve. The linearity was investigated by preconcentrating a series of standard solutions ranging from 0 to 500 µg L^−1^.

## Results and discussion

### Characterization of the nanocomposite

#### X-ray diffraction (XRD)

**Figure 1:**
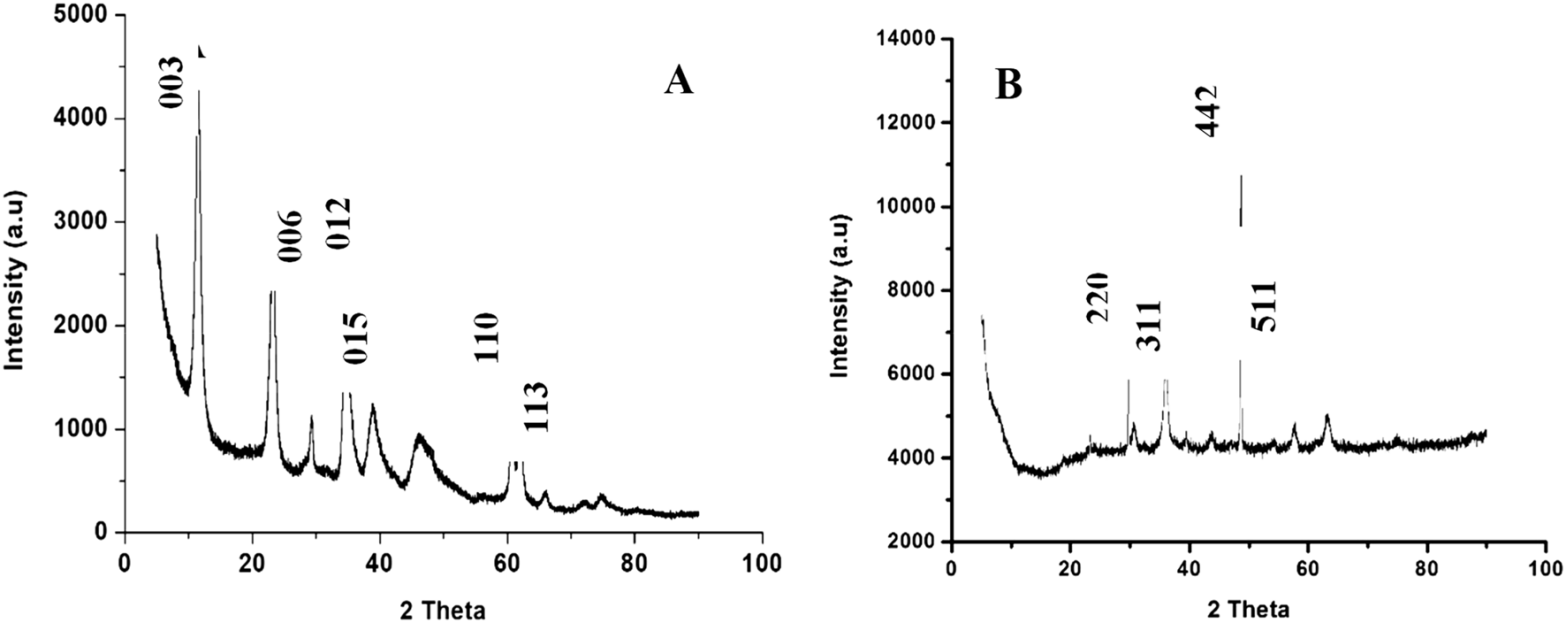
X-ray diffraction patterns of (**a**) magnetic Fe_3_O_4_/MgAl LDH nanocomposite, (**b**) MgAl-LDH.

Figure 1 presents the X-ray diffraction patterns of MgAl-LDH, Fe_3_O_4,_ and Fe_3_O_4_@MgAl LDH nanocomposite. The appearance of peaks at 2θ 11 (003), 24 (006), 35 (012), 40 (0 15), 47 (018), 62 (110), and 64 (113) in Fig. 1a was assigned to the interlayer spacing of Mg–Al-LDH present in the composite^7,33,34^. These peaks are the same as those observed in Fig. 1b. The peaks at theta values and Miller indices (in brackets) 20 (111), 30 (220), 38 (311), 43 (400), 55 (442), and 58 (511) confirmed the incorporation of Fe_3_O_4_ nanoparticles in the composite^31,35^. Similar peaks can be observed for pristine Fe_3_O_4_ nanoparticles. These findings proved that the prepared adsorbent was crystalline and they were similar to those reported elsewhere^35^.

#### Morphological properties and elemental composition of the nanocomposite

**Figure 2:**
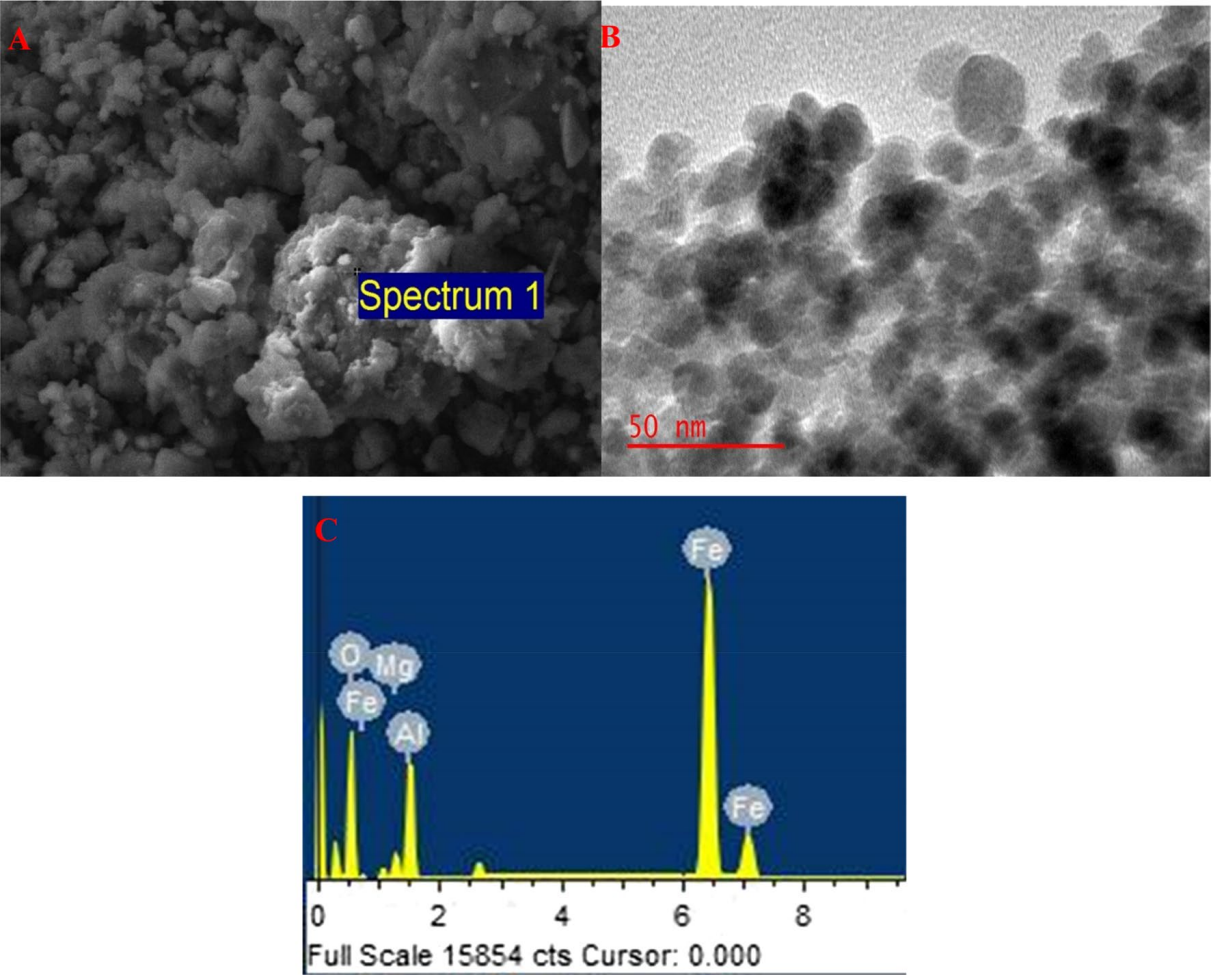
(**a**) SEM (10 µm) and (**b**) EDS and (**c**) TEM of Fe_3_O_4_@Al–Mg LDH.

The prepared magnetic Fe_3_O_4_@MgAl LDH was characterized by scanning electron microscope/(SEM/EDS) and transmission electron microscopy (TEM) to examine the morphological properties as well as elemental composition (Fig. 2a,b). The Fe_3_O_4_@MgAl LDH nanocomposite shows various sizes of particles which suggests that the adsorbent has several sorption active sites that can lead to higher adsorption capacities^30^. The EDS spectrum (Fig. 2c) shows that the nanocomposite was composed of Fe(31.58%), Mg(0.97%), Al(3.14%), and O(38.80%), confirming the successful synthesis of Fe_3_O_4_@MgAl LDH adsorbent and C(25.20%) which was from the carbon coating.

#### Brunauer–Emmett–teller (BET)

**Figure 3:**
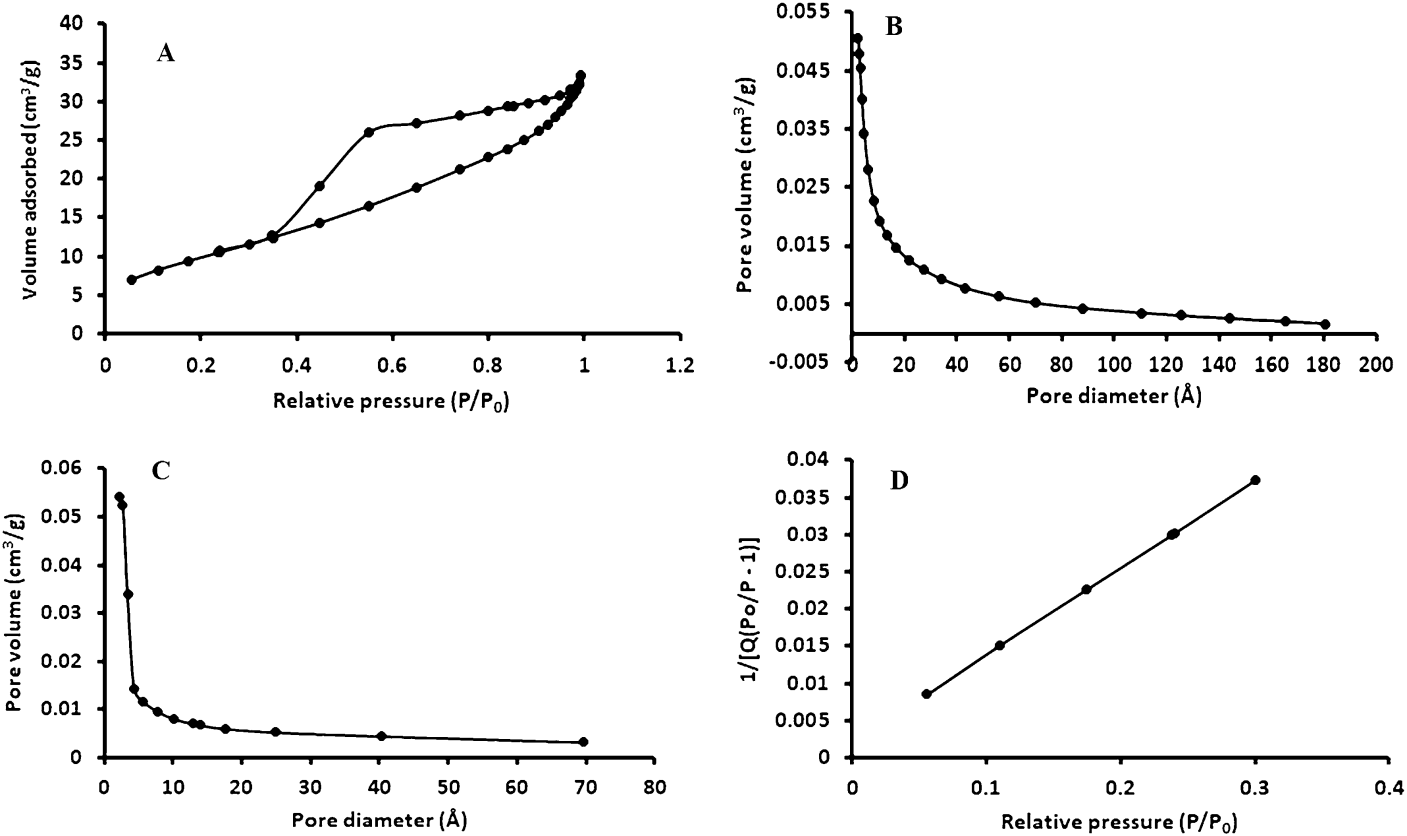
(**a**) N_2_ adsorption–desorption isotherm, (**b**,**c**) specific surface area pore volume and (**d**) pore size of the Fe_3_O_4_@MgAl LDH composite.

Nitrogen adsorption/desorption isotherm and the subsequent pore size distribution (obtained from the adsorption/desorption isotherm) for the Fe_3_O_4_@MgAl LDH composite are illustrated in Fig. 3. As shown in Fig. 3a, the curve has a distinct hysteresis loop that was observed in the pressure range of 0.4–1.0 p/p_0_ which is a typical type IV isotherm. This implied that the prepared composite was mesoporous. The BET specific surface area pore volume and pore size (Fig. 3b–d) of the mesoporous composite were 143m^2^/g, 0.049 cm^3^/g, and 5.34 nm, respectively. The BET surface areas for MgAl LDH and Fe_3_O_4_ were 101 and 36.5 m^2^/g, respectively. An increase in the surface area of the composite compared to Fe_3_O_4_ shows that the magnetic Fe_3_O_4_ was successfully coated with MgAl LDH. These results are comparable to the literature^7,24,33^ and they were better than those reported elsewhere^7,31^. The relatively high specific surface area proved that the prepared adsorbent is suitable for the adsorption of trace metals.

#### Fourier-transform infrared spectroscopy (FTIR)

**Figure 4:**
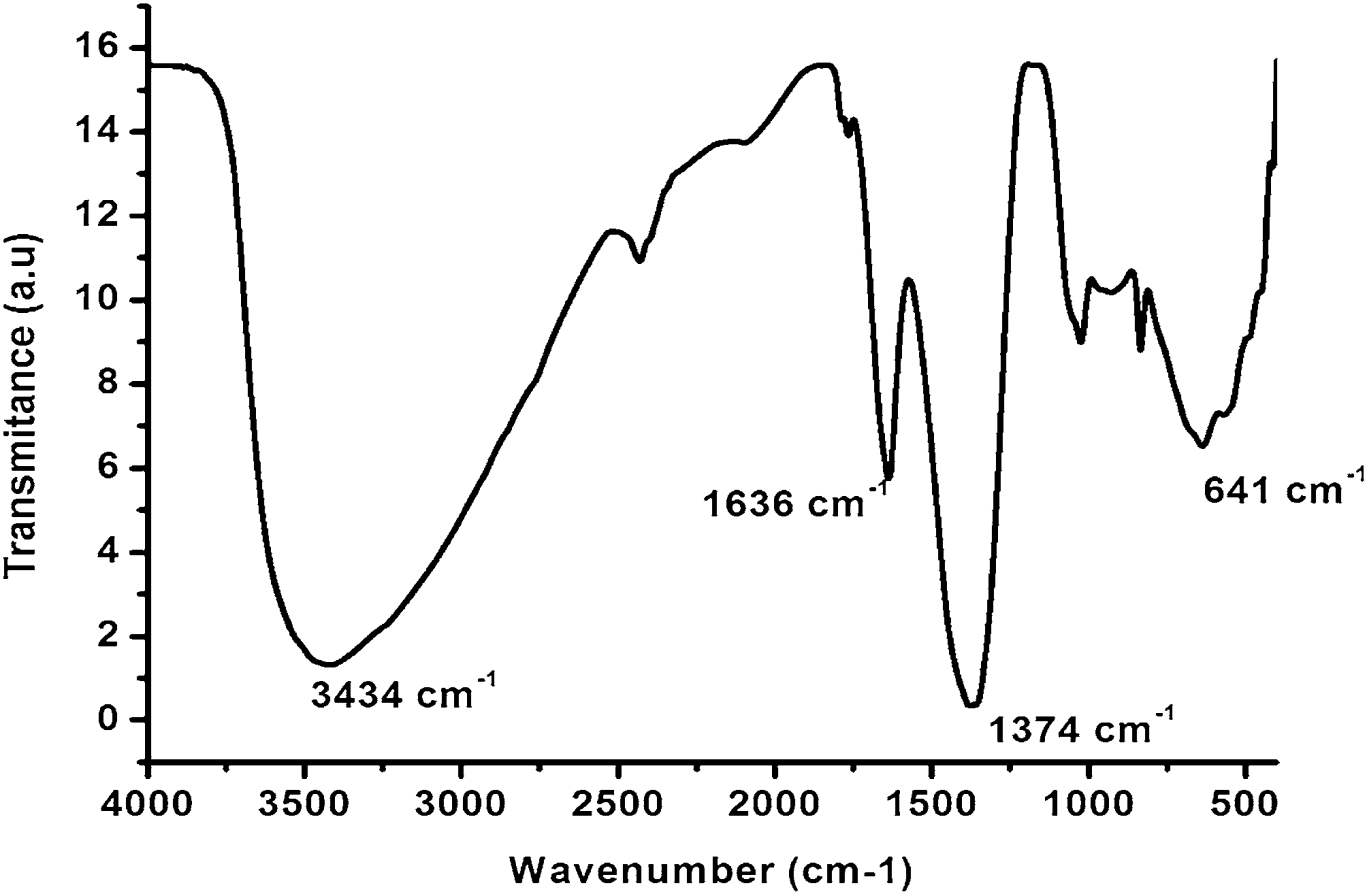
FTIR spectra of magnetic Fe_3_O_4_/MgAl LDH nanocomposite before and after adsorption.

The Fourier-transform infrared spectroscopy (FTIR) technique was used to assess the functional groups present on the surface of Fe_3_O_4_@MgAl-LDH nanocomposite and the results are presented in Fig. 4. The broad peak at 3434 cm^−1^ was ascribed to O–H stretching of LDH layers and interlayer water molecules^7,33,36^. A sharp at 1374 cm^−1^ was assigned to the stretching vibration of intercalated –NH_3_ from the synthesis of magnetite. The vibrations at 1636 cm^−1^, 783 cm^−1^, and 641 cm^−1^ were ascribed to the presence of CO_3_^−^ ions in the interlayer of LDHs^36^. The results are comparable to the literature^31,36^.

### Selection of the adsorbent

**Figure 5:**
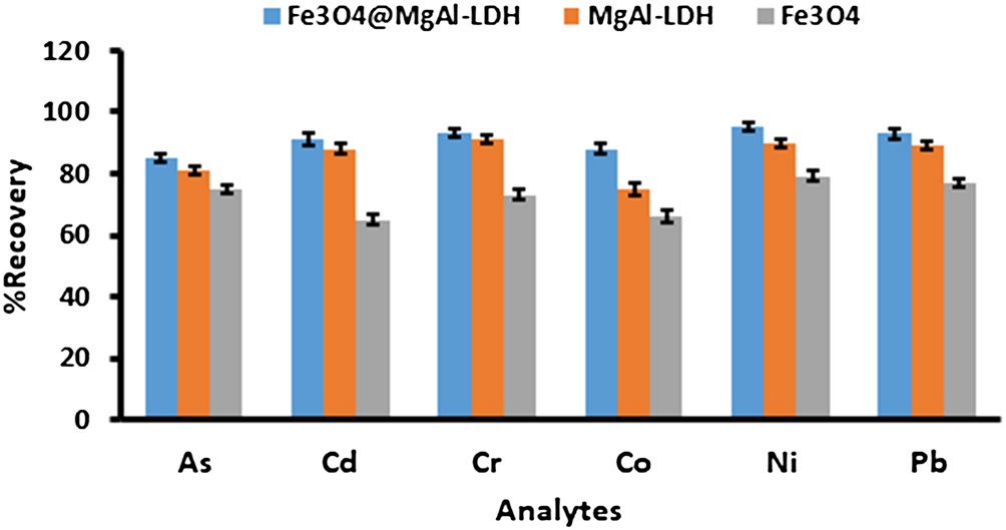
Selection of an adsorbent: experiment condition: mass of adsorbent 50 mg; sample pH 7.5; extraction time 10 min; eluent concentration 1.0 mol L^−1^.

The selection of a suitable and effective adsorbent is one of the critical aspects of the extraction and preconcentration of trace metals. Therefore, in this study, the sorption and extraction ability of Fe_3_O_4_/MgAl LDH nanocomposite was evaluated in comparison with Fe_3_O_4_ and MgAl-LDH adsorbents. Figure 5 demonstrates that the Fe_3_O_4_/MgAl LDH nanocomposite and MgAl LDH exhibited better extraction efficacy than Fe_3_O_4_ nanoparticles. However, due to magnetism, Fe_3_O_4_/MgAl LDH nanocomposite displayed better separability compared to MgAl-LDH. Thus, the Fe_3_O_4_/MgAl LDH nanocomposite was chosen as the adsorbent for the preconcentration of trace metals.

### Optimization of the preconcentration procedure using a central composite design

**Figure 6:**
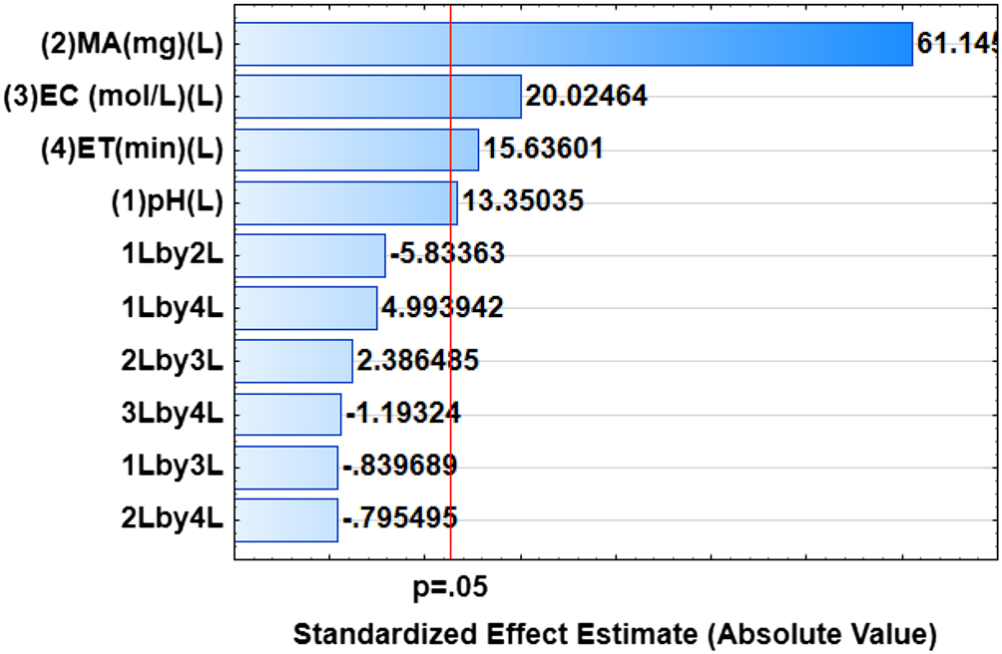
Pareto charts of standardized effects for optimization of preconcentration procedure.

The CCD matrix and respective recoveries (analytical response) for each analyte are presented in supplementary data (Table S1). Analysis of variance (ANOVA) was carried to investigate the quality of the RSM model and assess the most significant parameters as well as to examine the interactions between the independent variables. The ANOVA results reproduced in the form of a Pareto chart (Fig. 6) revealed that the p-values of the individual variables were less than 0.05, implying that they are significant at a 95% confidence level.

#### Response surface methodology

**Figure 7:**
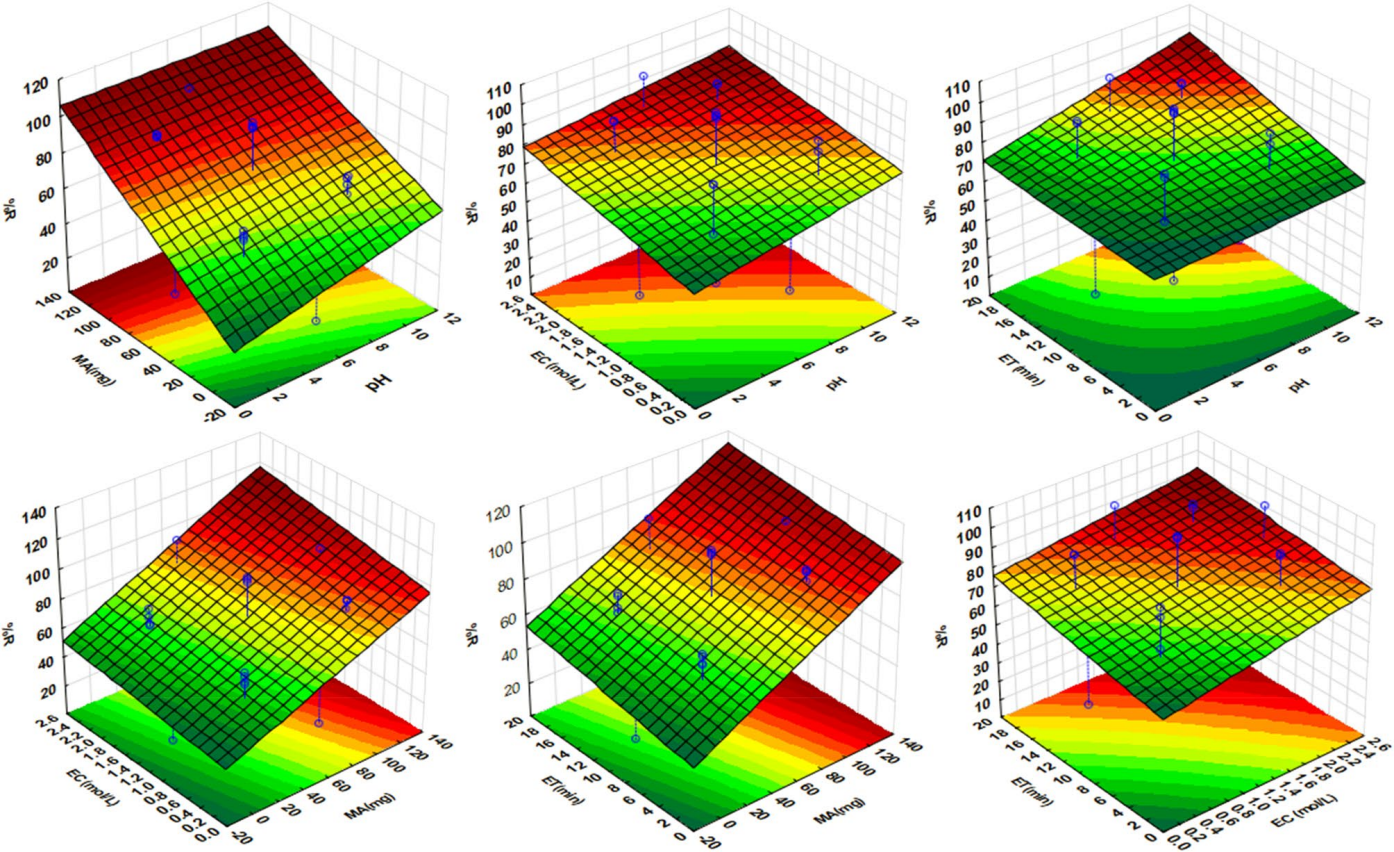
Three-dimension response surface plots for optimization of the independent variables.

The three-dimensional response surface plots of the second-order polynomial equation were used to examine interactions between independent variables and their combined effect on the analytical response. The combined effect of two variables was assessed by varying them within the determined ranges while keeping two other variables at zero level (central point) (Fig. 7). The response surface plots for the effect of pH and eluent concentration, pH and extraction time, and pH and mass of adsorbent on the preconcentration of target analytes show that the % recovery increased with a slight increase in sample pH. These observations agreed with the Pareto chart illustration which demonstrated that sample pH was ranked as the least significant factor. The response surface plots showing the effect of the mass of adsorbent and pH, the mass of adsorbent and eluent concentration as well as the mass of adsorbent and extraction time revealed that the analytical response for the target analytes increased with an increase in the mass of adsorbent. This might be due to the availability of adsorption sites on the surface of the adsorbent which enhances the extraction efficiency of the adsorbent. Lastly, the increase in extraction time and eluent concentration also proved to have a positive effect on the analytical response.

#### Optimization by desirability function

**Figure 8:**
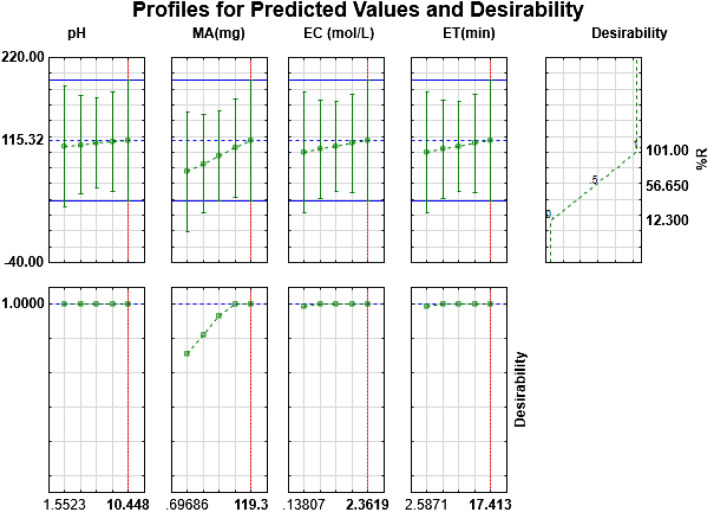
Profile for the predicted values and desirability function for the removal percentages of As(III), Cd(II), Cr(III), Co(II), Ni(II), and Pb(II) ions.

The desirability function was employed to optimize individual variables (sample pH, extraction time, eluent concentration, and mass of adsorbent) that affect the pre-concentration process. The desirability values of 0.0, 0.5, and 1.0 corresponded to the minimum, middle and maximum functions. The values in the desirability profile (Fig. 8) that are closer to 1.0 imply that the corresponding parameter or variable condition is optimum^37^. The optimum conditions for simultaneous preconcentration of As(III), Cd(II), Cr(III), Co(II), Ni(II), and Pb(II) ions were selected to be 6.5, 84 mg, 10 min, and 2.0 mol L^−1^ for sample pH, mass of adsorbent, extraction time and eluent concentration, respectively.

### Adsorption capacity

The adsorption capacity of the nanocomposite was investigated under optimum conditions. An aliquot of 100 mL synthetic solutions containing 10–250 mg L^−1^ of As(III), Cd(II), Cr(III), Co(II), Ni(II), and Pb(II) ions was processed using the optimized preconcentration method. After equilibrium has been reached the solutions were determined using ICP-OES. The adsorption capacity was measured by using the following formula:

4 $$q_{e} = \frac{{\left( {C_{0} - C_{e} } \right)V}}{m}$$

where q_e_ is the adsorption capacity, C_0_ and C_e_ are the initial and equilibrium concentrations of the analytes, m and V are the mass of adsorbent and volume of synthetic solution. The equilibrium data were fitted to nonlinear Langmuir and Freundlich equations (Eqs. 5, 6). The parameters together with the correlation coefficient that resulted from the fitting of the two models are listed in Table 1.

**Table 1:** Non-linear adjusted Langmuir and Freundlich isotherm parameters.

Analytes	Langmuir					Freundlich				
	q_max_ (mg g^−1^)	K_L_ (L g^−1^)	R^2^	Adj R^2^	RSE	K_F_ (L/mg)	n	R^2^	Adj R^2^	RSE
As	124 ± 1	0.078 ± 0.003	0.9675 ± 0.02	0.9280 ± 0.03	10.2 ± 0.5					
Cd	122 ± 4	0.051 ± 0.002	0.9602 ± 0.03	0.9354 ± 0.05	9.1 ± 0.1	24.6 ± 0.4	3.09 ± 0.03	0.8638 ± 0.01	0.7144 ± 0.03	21.1 ± 0.3
Co	138 ± 3	0.045 ± 0.001	0.9648 ± 0.06	0.9234 ± 0.02	9.7 ± 0.3	22.6 ± 0.2	2.81 ± 0.02	0.9204 ± 0.02	0.8279 ± 0.02	17.2 ± 0.1
Cr	116 ± 4	0.046 ± 0.002	0.9638 ± 0.04	0.9256 ± 0.01	11.2 ± 0.6	21.6 ± 0.1	3.08 ± 0.03	0.8885 ± 0.02	0.7631 ± 0.01	16.9 ± 0.2
Ni	120 ± 1	0.042 ± 0.003	0.9077 ± 0.02	0.8921 ± 0.01	10.7 ± 0.6	23.5 ± 0.3	3.19 ± 0.08	0.8670 ± 0.01	0.7207 ± 0.02	19.1 ± 0.4
Pb	130 ± 4	0.061 ± 0.007	0.9834 ± 0.07	0.9412 ± 0.02	8.3 ± 0.1	25.2 ± 0.6	3.06 ± 0.03	0.9131 ± 0.04	0.8131 ± 0.03	13.2 ± 0.2

5 $${{\text{q}}}_{{\text{e}}}=\frac{{{\text{q}}}_{{\text{max}}}{{\text{K}}}_{{\text{L}}}{{\text{C}}}_{{\text{e}}}}{1+{{\text{K}}}_{{\text{L}}}{{\text{C}}}_{{\text{e}}}}$$

6 $${{\text{q}}_{\text{q}}}={{\text{K}}_{\text{F}}}{{\text{C}}_{\text{e}}^{1/\text{n}}}$$

where q_e_: amount adsorbed; q_max_: maximum monolayer adsorption; K_L_: Langmuir constant; C_e_; concentration of adsorbate at equilibrium; RL: separation factor; K_F_: adsorption capacity; 1/n: adsorption intensity.

The adsorption isotherms of the Fe_3_O_4_@MgAl LDH for As, Cd, Co, Cr, Ni, and Pb were performed under the optimized conditions. The results are shown in Table 1. As reported in Table 1, the R^2^ and RSE values of Langmuir models were higher than the R^2^ and RSE values of Freundlich. This suggested that the adsorption processes conformed well to the Langmuir model. The experimental maximum adsorption capacities for As, Cd, Co, Cr, Ni, and Pb Langmuir isotherms were found to be (124, 122, 138, 116, 120, 130) and for Freundlich were 0, 24.6, 22.6, 21.6, 23.5 and 25.2).

#### Adsorption mechanism

**Figure 9:**
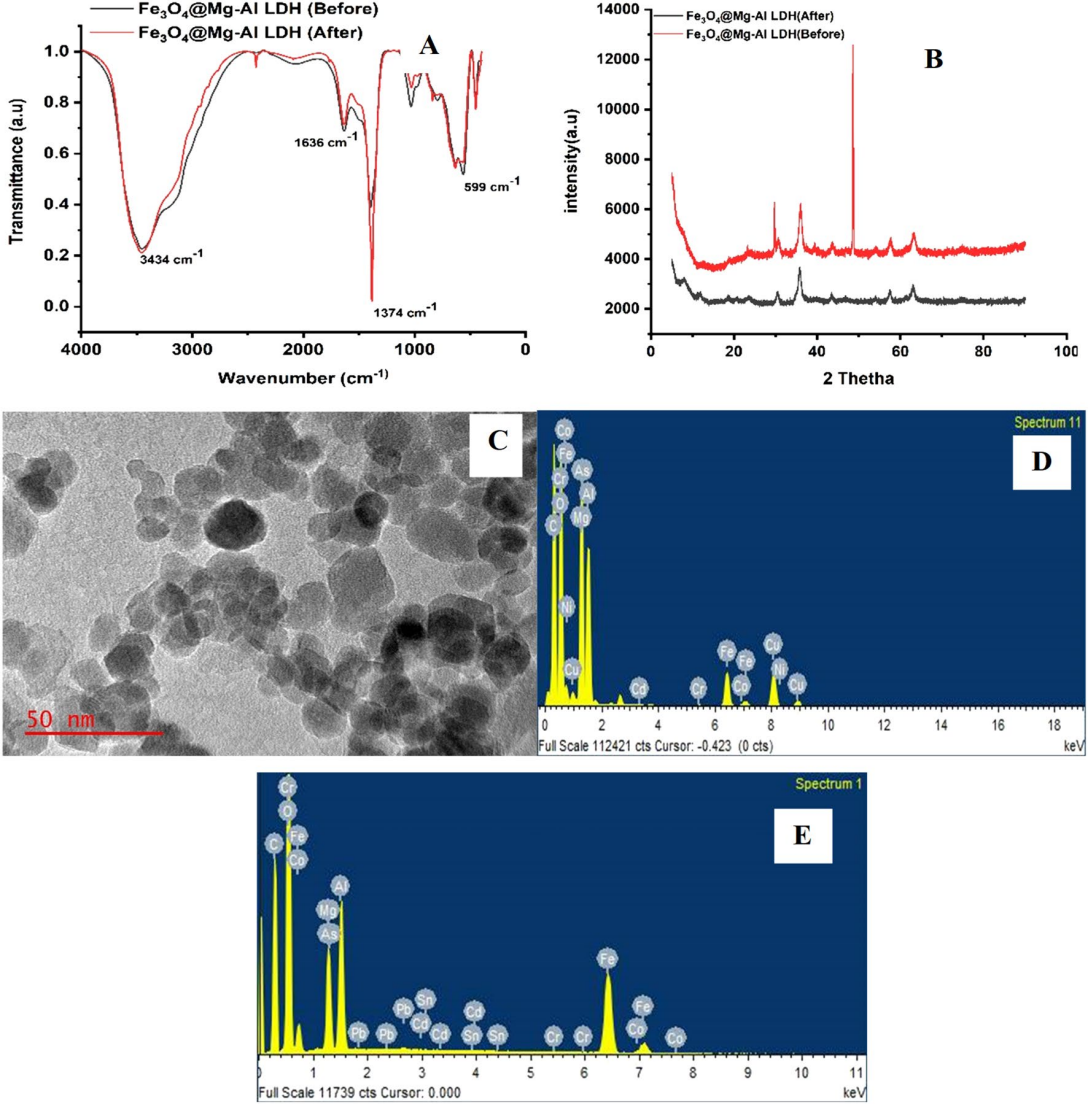
(**a**) FTIR spectra (**b**) XRD (**c**) TEM image and (**d**,**e**) EDS spectra.

Even though the FTIR spectra of Fe_3_O_4_/MgAl LDH nanocomposite before and after adsorption of As(III), Cd(II), Cr(III), Co(II), Ni(II), and Pb(II) (Fig. 9a) showed no noticeable changes. The XRD patterns, EDS, and TEM spectra respectively were used for clarifying the removal mechanisms (Fig. 9b–d). It can be observed from XRD patterns demonstrated by (Fig. 9b) showed high crystallinity of the compounds before and after adsorption. The typical diffraction peaks of the Fe_3_O_4_@MgAl LDH before adsorption and after adsorption were still strong and sharp. The EDX of Fe_3_O_4_@MgAl LDH nanocomposite after adsorption shows (Fig. 9d,e) it is mainly composed of the elements O, Fe, Mg, Al, As, Ni, Cd, Cr, Co, and Pb this confirms the adsorption of the analytes by the adsorbent was successful.

**Figure 10:**
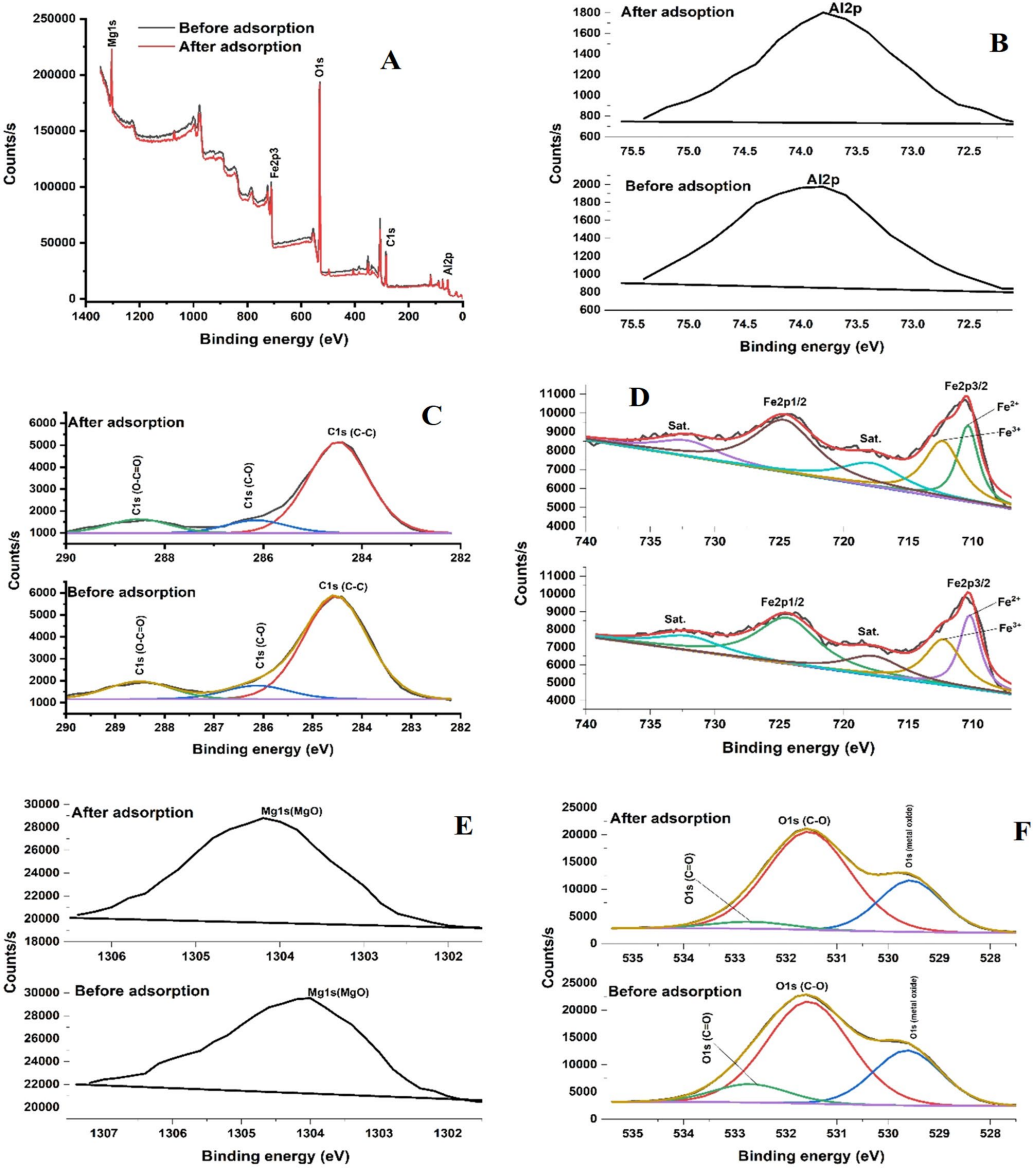
(**a**–**f**) XPS of Fe_3_O_4_@Al–Mg LDH after adsorption.

The survey spectra of Fe_3_O_4_@MgAl LDH nanocomposites confirm the existence of Al, Mg, Fe, O, and C atoms on the nanocomposites before and after adsorption. Noticeably, the survey spectra of Fe_3_O_4_@MgAl LDH nanocomposite after the adsorption of metal ions show an increase in the intensity signal as compared to before adsorption, thus suggesting the conceivable adsorption of metal ions in solution (Fig. 10a)^38^. Subsequently, the high-resolution XPS spectra of Fe2p (Fig. 10d) exhibited two main peaks at 711.25 and 724.41 eV corresponding to Fe2p3/2 and Fe2p1/2, respectively. The deconvoluted Fe2p spectra before and after adsorption displayed Fe^2+^ and Fe^3+^ at 710.24 and 712.27 eV confirming the existence of Fe_3_O_4_ in the nanocomposite^19^. No significant shifts in the peaks on Fe2p were observed considering the binding energy before and after adsorption, indicating that Fe_3_O_4_ retained its chemical structure on the composite. As expected, the high-resolution spectra of Al2p (Fig. 10b) and Mg1s (Fig. 10e) with binding energies of 73.8 and 1304.2 eV show negligible changes before and after adsorption.

The deconvoluted high-resolution spectra of C1s (Fig. 10c) shows three-carbon contributions corresponding to the C–C, C–O and O–C=O at binding energies of 284.5, 286.1 and 288.5, respectively, before and after adsorption. Subsequently, the C1s signal increased after adsorption from 22.5 to 23.9%. The increase in the C1s signal can be subjected to the increase in interlayer CO_3_^2−^ anions^39^. Moreover, the increase in the C1s signal correlates with the reduction in the O1s signal, as the reduction on the surface hydroxyl groups was complemented by the increase on the intercalated CO3^2−^ anions, which is one of the adsorption sites that can effectively adsorb metal cations of interest^39–41^. Interestingly, the Ols (Fig. 10f) content decreased after adsorption, indicating possible adsorption of As, Pb, Cd, Cr, Co, and Ni metals through the interaction with the present hydroxyl groups on the surface of the composite, which corroborates with the EDS results. Although adsorption primarily occurs through the interaction of the analyte with the surface hydroxyl groups, another site of adsorption concerning the intercalated CO_3_^2−^ has been reported^40^. This implies the adsorption of As, Pb, Cd, Cr, Co, and Ni occurs on both the intercalated MgAl-CO_3_^2−^ or on the surface MgAl-OH_x_/deprotonated MgAl-O^−^^40^. To this end, the possible mechanisms of adsorption related to these interactions are listed as follows:

### Analytical figures of merit and method validation

Analytical figures of merit of the UASPME/ICP-OES method, such as linear range, correlation coefficient (R^2^), preconcentration factor (PF), enhancement factor (EF), precision (intraday and interday), LODs, and LOQs were determined as discussed in “Experimental” section for the target analytes. The calibration curve equations, linearity, R^2^, and the enrichment factors for each analyte are presented in Table 2.

**Table 2:** Calibration curve equations, correlation coefficients and enrichment factors of UASPME/ICP-OES method for As(III), Cd(II), Cr(III), Co(II), Ni(II), and Pb(II) ions.

Analytes	Equation before preconcentration	Equation after preconcentration	R^2^	Enrichment factor (EF)^a^
As(III)	y = 0.0104 (± 0.0005)x + 0.0027 (± 0.0004)	y = 0.783 (± 0.0093)x + 0.0015 (0 ± .0002)	0.9997 ± 0.0009	75.3 ± 0.1
Cd(II)	y = 0.0202 (± 0.009)x + 0.0045 (± 0.007)	y = 0.618 (± 0.008)x + 0.0022 (± 0.0004)	0.9991 ± 0.0012	60.6 ± 0.3
Co(II)	y = 0.0135 (± 0.0011)x + 0.0055 (± 0.0008)	y = 0.955 (± 0.005)x + 0.0102 (± 0.0003)	0.9989 ± 0.0007	70.7 ± 0.5
Cr(III)	y = 0.0143 (± 0.0006)x + 0.0072 (± 0.0011)	y = 0.737 (± 0.0009)x + 0.0010 (± 0.007)	0.9992 ± 0.0005	51.5 ± 0.4
Ni(II)	y = 0.0307 (± 0.0013)x + 0.0089 (± 0.0011)	y = 0.936 (± 0.001)x + 0.0115 (± 0.003)	0.9994 ± 0.0009	87.5 ± 0.3
Pb(II)	y = 0.0122 (± 0.0009)x + 0.0033 (± 0.0005)	y = 0.884 (± 0.003)x + 0.0011 (± 0.0005)	0.9987 ± 0.0005	72.5 ± 0.4

^a^Enrichment factor was defined as the ratio between the slope of the calibration curves before and after preconcentration.

The preconcentration factor, calculated as the ratio of the sample volume (100 mL) divided by the eluent volume (2.5 mL), was found to be 40. The LODs and LOQs of the UASPME/ICP-OES method are illustrated in Table 3. The precision (expressed (%RSD)) of the method was investigated in terms of intraday (repeatability, n = 10) and interday (reproducibility n = 5 working day). The %RSD for the analysis of As, Cd, Co, Cr, Ni, and Pb by the UASPME/ICP-OES method were less than 5% (Table 3).

**Table 3:** Analytical performances of UA-DSPME/ICP-OES method.

Analytes	As(III)	Cd(II)	Co(II)	Cr(III)	Ni(II)	Pb(II)
Linearity (μg L^−1^)	0.2–350	0.3–450	0.2–400	0.2–500	0.3–450	0.3–350
LOD (μg L^−1^)	0.11 ± 0.01	0.21 ± 0.02	0.16 ± 0.01	0.15 ± 0.02	0.22 ± 0.03	0.17 ± 0.02
LOQ (μg L^−1^)	0.35 ± 0.03	0.70 ± 0.07	0.55 ± 0.03	0.50 ± 0.07	0.73 ± 0.10	0.57 ± 0.07
Intraday (%RSD)	2.3	1.6	1.8	1.3	1.9	2.5
Interday (%RSD)	4.3	2.5	3.6	3.9	4	4.6

Table 4 displays the results obtained for the determination of As(III), Cd(II), Cr(III), Co(II), Ni(II) and Pb(II) from NIST SRM 1643e (trace elements in water), CRM ERM-CA713 (trace metals in wastewater) and NIST SRM 1640a (trace elements in natural water). The relative error (%RE) values ranged from − 2.4 to 1.7%, signifying that the developed method had high accuracy. Additionally, the obtained values agreed with the certified values at a 95% confidence level.

**Table 4:** Determination of As(III), Cd(II), Cr(III), Co(II), Ni(II) and Pb(II) ions (concentration, μg L^−1^) in certified and standard reference materials.

Analytes	ERM^®^-CA713			SRM 1640a			SRM 1643e		
	Certified	Found	%RE	Certified	Found	%RE	Certified	Found	%RE
As(III)	10.8 ± 0.3	10.5 ± 0.5	− 2.8	8.08 ± 0.07	8.01 ± 0.08	− 0.87	60.5 ± 0.6	61.1 ± 1.1	0.99
Cd(II)	5.09 ± 0.20	4.98 ± 0.15	− 0.22	3.99 ± 0.07	4.01 ± 0.05	0.50	6.57 ± 0.07	6.62 ± 0.05	0.76
Cr(III)	20.9 ± 0.1.3	20.7 ± 0.9	− 0.96	40.5 ± 0.3	40.9 ± 0.9	0.99	20.4 ± 0.2	20.6 ± 0.5	0.98
Co(II)	–			20.2 ± 0.2	19.8 ± 0.4	− 2.0	27.8 ± 0.3	28.1 ± 0.7	1.1
Ni(II)	50.3 ± 1.4	51.2 ± 2.3	1.7	25.3 ± 0.1	24.9 ± 0.5	− 1.6	62.4 ± 0.7	62.6 ± 0.6	0.32
Pb(II)	49.7 ± 1.7	50.3 ± 2.1	1.2	12.0 ± 0.1	11.9 ± 0.3	− 0.83	19.6 ± 0.2	19.3 ± 0.5	− 1.5

Additionally, the validity and applicability of the developed UASPME/ICP-OES procedure were evaluated by analysis of As(III), Cd(II), Cr(III), Co(II), Ni(II), and Pb(II) ions in complex matrices such tap and river water. The accuracy of the method was evaluated by spiking the water samples with 10 µg L^−1^ of the target analytes the analytical results are presented in Table 5. As can be seen, the recoveries for the trace elements in each type of water sample were above 95% standard deviations less than 3%.

**Table 5:** Analysis of real samples using DSPME-SAE/ICP-OES.

Analytes	Added (μg L^−1^)	Tap water		River water	
		Found (μg L^−1^)	Recovery (%)	Found (μg L^−1^)	Recovery (%)
As(III)	0	BLOD	–	0.53 ± 0.03	–
	10	9.87 ± 0.11	98.7	10.5 ± 0.3	99.2
Cd(II)	0	BLOD	–	0.87 ± 0.01	–
	10	9.78 ± 0.08	97.8	10.7 ± 0.7	98.3
Co(II)	0	5.77 ± 0.06	–	16.3 ± 0.9	–
	10	15.7 ± 0.2	99.3	26.2 ± 0.8	99.1
Cr(III)	0	13.6 ± 0.9	–	23.8 ± 0.3	–
	10	23.5 ± 0.9	98.6	33.7 ± 0.7	99.4
Ni(II)	0	25.8 ± 0.8	–	56.7 ± 0.3	–
	10	35.8 ± 0.5	99.8	66.9 ± 0.8	102
Pb(II)	0	2.37 ± 0.10	–	6.23 ± 0.05	–
	10	12.3 ± 0.6	99.3	16.1 ± 0.9	98.7

### Analysis of water samples using UASPME/ICP-OES method

The developed UASPME/ICP-OES method was applied to the determination of trace As(III), Cd(II), Cr(III), Co(II), Ni(II), and Pb(II) ions in acid mine drainage effluents (SB, WB and Site 3) and the results are shown in Table 6. These findings suggested that the proposed method had the capability to extract, preconcentrate, and determine trace metals ions in real samples with complex matrices.

**Table 6:** Recoveries of As(III), Cd(II), Cr(III), Co(II), Ni(II) and Pb(II) ions from AMD water samples using the proposed UASPME/ICP-OES method, n = 3.

Samples	Elemental concentration in μg L^−1^, n = 3					
	As(III)	Cd(II)	Cr(III)	Co(II)	Ni(II)	Pb(II)
SB	ND	0.695	2.43	46.9	2.30	ND
WB	74.9	ND	2.83	48.93	ND	ND
Site 3	8.69	0.175	21.5	41.0	ND	0.950

The comparison of analytical characteristics of UASPME/ICP-OES method for preconcentration of As(III), Cd(II), Cr(III), Co(II), Ni(II), and Pb(II) ions with previously researched preconcentration procedures specifically (solid-phase based extraction) reported in the literature are summarised in Table 7. It was observed that the proposed method had superior LOD, EF, RSD, and linear range compared to those reported elsewhere^42,43^. In the addition, parameters such as liner calibration range, LOD, EF, and RSD for UASPME/ICP-OES technique were comparable to those stated in the literature^44^. However, the analytical performance on the developed method was relatively poor than those reported by Refs^32,45^.

**Table 7:** Analytical characteristics of UA-DSPME/ICP-OES in comparison with other methods in the literature.

Analytes	Analytical method	Linearity µg L^−1^	LOD µg L^−1^	%RSD	EF	Refs
Cr, Co, Cd, Zn, and Pb	mag-SPE/ICP-MS		0.001–0.11	< 10		^46^
Cd (II)	MSPE/ETAAS	0.005–0.715	0.0019	2.8		^23^
Pb	SPE/FAAS	1.7–1000	1.7	1.3	550	^47^
Cd, Pb, Ni	SPE/FAAS		0.15, 0.40, 0.8	< 8.5		^48^
Cd(II)	VA-DLLME/FAAS		0.25	< 2.5		^43^
Cd, Cr, Cu, and Pb	SPE/EDXRF		24, 2.8, 16 and 9.7	2–8.1		^42^
Co, Ni, and Cd	DMSPE/ICP-MS		0.0001, 0.0012 and 0.00009			^45^
Pb	MSPE /FAAS		0.2	3.8	200	^44^
Pb	UA-MSPE/ICP-OES	0.1–500	0.023	1.6	90	^32^
As, Cd, Cr, Pb, Co, Ni	UA-DSPME/ICP-OES	0.2–500	0.11–0.22	1.3–2.5	51.5–87.5	This work

### Regeneration studies

**Figure 11:**
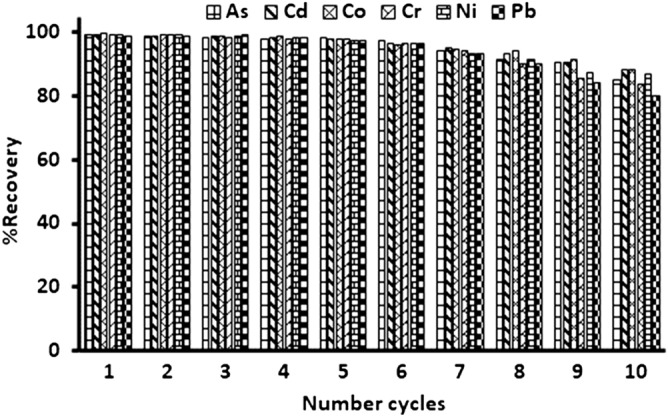
% recoveries of each investigated metal ion.

The reusability and regeneration of Fe_3_O_4_@MgAl LDHs composite was performed by conducting subsequent adsorption/desorption experiments. Nitric acid (2.0 mol L^˗1^) was used to regenerate the spent Fe_3_O_4_@MgAl LDHs composite. The amount of trace metals after every adsorption/desorption cycle was expressed in terms of percentage recovery (%R). Figure 11. illustrate the % recoveries of each investigated metal ion. As shown in Figure, the Fe_3_O_4_@MgAl LDHs composite retained its adsorption capabilities up to the sixth cycle. After the sixth cycle, the adsorption efficiency gradually decreased by a small margin. At the end of the eighth adsorption/desorption cycle, the %R were 85.3%, 88.1%, 83.6%, 79.9%, 88.5% and 86.7% for As, Cd, Cr, Pb, Co, and Ni, respectively. Nonetheless, it can be concluded that Fe_3_O_4_@MgAl LDHs composite had demonstrated exceptional regeneration and reusability as the percentage recoveries decrease only slightly after the sixth cycle. Furthermore, the study of the stability of the prepared magnetic Fe_3_O_4_@MgAl LDH adsorbent in the adsorption system was performed by testing for the presence of Fe, Mg, and Al to evaluate the long-term stability. There were no Fe, Mg, and Al detected in all the ten parallel cycles, indicating there was no leaching of Fe_3_O_4_@MgAl LDH. Also, the prepared adsorbent showed stable magnetic property before and after use.

## Conclusion

The Fe_3_O_4_@MgAl LDH composite was prepared successfully via the co-precipitation method. The prepared composite was characterized through instruments such as SEM–EDS, TEM, BET surface area, and FTIR. The Fe_3_O_4_@MgAl LDH composite was applied as an adsorbent in UA-DSPME for preconcentration of trace As(III), Cd(II), Cr(III), Co(II), Ni(II), and Pb(II) ions in river water, tap water, and mine wastewater samples. Experimental parameters affecting UA-DSPME/ICP-OES method were optimized using a multivariate approach. The UA-DSPME/ICP-OES method revealed outstanding analytical characteristics such as simplicity, rapidity, low LODs, high accuracy, enrichment factors, and precision. Furthermore, the performance of the UA-DSPME/ICP-OES method was applied for the determination of trace metals in complex matrices.

## Supplementary Information

Supplementary Information.
