# Supplementary material for: Magnetic Fe3O4@Mg/Al-layered double hydroxide adsorbent for preconcentration of trace metals in water matrices
Source: Sci Rep. 2021 Jan 27;11:2302. doi: 10.1038/s41598-021-81839-8 (PMC7840964; doi:10.1038/s41598-021-81839-8)
Supplement: Supplementary file 1 — Supplementary Information. [file 41598_2021_81839_MOESM1_ESM.doc]

**Magnetic Fe_3_O_4_@Mg/Al-layered double hydroxide adsorbent for preconcentration of trace metals in water matrices**

Luthando Nyaba^a,b^, Tshimangadzo S. Munonde^a,b^, Anele Mpupa^a,b^, Philiswa N. Nomngongo^a,b,c^

*^a^Department of Chemical Sciences, University of Johannesburg, Doornfontein Campus, P.O. Box 17011, Doornfontein, 2028, South Africa*

*^b^DSI/NRF SARChI Chair: Nanotechnology for Water, University of Johannesburg, Doornfontein, 2028, South Africa*

*^c^DSI/Mintek Nanotechnology Innovation Centre, University of Johannesburg, Doornfontein, 2028, South Africa*

Supplementary data

Table S1 design matrix and analytical response

|  | **Parameters** | | | | **% Recoveries** | | | | | |
| --- | --- | --- | --- | --- | --- | --- | --- | --- | --- | --- |
| **Standard Run** | pH | MA (mg) | EC (mol/L) | ET (min) | As | Cd | Co | Cr | Ni | Pb |
| **1** | 3 | 20 | 0.5 | 5 | 63.3 | 64.6 | 61.9 | 67.9 | 61.9 | 56.2 |
| **2** | 3 | 20 | 0.5 | 15 | 65.5 | 66.1 | 63.4 | 72.2 | 73.4 | 62.6 |
| **3** | 3 | 20 | 2 | 5 | 63.9 | 69.0 | 62.7 | 68.7 | 72.7 | 69.8 |
| **4** | 3 | 20 | 2 | 15 | 64.8 | 63.4 | 67.8 | 89.0 | 67.8 | 67.1 |
| **5** | 3 | 100 | 0.5 | 5 | 86.5 | 90.9 | 84.9 | 81.6 | 84.9 | 87.3 |
| **6** | 3 | 100 | 0.5 | 15 | 85.2 | 91.3 | 86.6 | 90.5 | 86.6 | 82.5 |
| **7** | 3 | 100 | 2 | 5 | 90.3 | 92.6 | 90.1 | 87.5 | 90.1 | 92.0 |
| **8** | 3 | 100 | 2 | 15 | 92.5 | 92.7 | 93.5 | 88.2 | 93.5 | 94.1 |
| **9** | 9 | 20 | 0.5 | 5 | 63.2 | 69.9 | 69.2 | 67.0 | 69.2 | 67.3 |
| **10** | 9 | 20 | 0.5 | 15 | 70.3 | 72.8 | 62.4 | 69.3 | 68.4 | 70.7 |
| **11** | 9 | 20 | 2 | 5 | 65.8 | 63.9 | 61.2 | 67.7 | 68.2 | 66.7 |
| **12** | 9 | 20 | 2 | 15 | 73.7 | 74.6 | 76.7 | 80.6 | 76.7 | 76.3 |
| **13** | 9 | 100 | 0.5 | 5 | 88.8 | 85.7 | 87.0 | 88.9 | 78.6 | 88.0 |
| **14** | 9 | 100 | 0.5 | 15 | 90.1 | 91.3 | 89.7 | 90.6 | 92.6 | 90.3 |
| **15** | 9 | 100 | 2 | 5 | 92.3 | 91.2 | 89.9 | 89.2 | 86.9 | 91.7 |
| **16** | 9 | 100 | 2 | 15 | 89.1 | 91.0 | 92.3 | 89.7 | 93.5 | 0.7 |
| **17** | 1.55 | 60 | 1.25 | 10 | 26.5 | 23.7 | 23.6 | 21.3 | 23.6 | 22.0 |
| **18** | 10.4 | 60 | 1.25 | 10 | 55.6 | 63.1 | 65.2 | 66.6 | 67.0 | 65.3 |
| **19** | 6 | 0.7 | 1.25 | 10 | 10.4 | 12.3 | 11.8 | 10.6 | 10.8 | 9.4 |
| **20** | 6 | 119 | 1.25 | 10 | 100 | 100 | 103 | 101 | 102 | 104 |
| **21** | 6 | 60 | 0.14 | 10 | 37.1 | 33.4 | 44.7 | 42.8 | 44.7 | 36.0 |
| **22** | 6 | 60 | 2.36 | 10 | 99.4 | 98.5 | 100 | 99.7 | 100 | 99.7 |
| **23** | 6 | 60 | 1.25 | 2.59 | 55.3 | 54.7 | 52.8 | 57.9 | 60.1 | 66.3 |
| **24** | 6 | 60 | 1.25 | 17.4 | 99.7 | 98.2 | 97.2 | 96.3 | 97.2 | 97.7 |
| **25 (C)** | 6 | 60 | 1.25 | 10 | 96.0 | 99.4 | 99.6 | 102 | 99.6 | 99.0 |
| **26 (C)** | 6 | 60 | 1.25 | 10 | 95.8 | 101 | 99.2 | 102 | 99.2 | 99.3 |
